# Enhanced Prediction of Atrial Fibrillation in Patients With Ischemic Stroke Through Electronic Medical Records and Text Mining: Algorithm Development and Validation

**DOI:** 10.2196/78117

**Published:** 2026-03-10

**Authors:** Yu-Wei Chen, Sheng-Feng Sung, Ya-Han Hu, Yu-Hsuan Yang

**Affiliations:** 1 Department of Neurology, Landseed International Hospital, Taoyuan City, Taiwan; 2 Department of Neurology, National Taiwan University Hospital, Taipei, Taiwan; 3 Center for General Education, National Central University, Taoyuan City, Taiwan; 4 Division of Neurology, Department of Internal Medicine, Ditmanson Medical Foundation Chia-Yi Christian Hospital, Chiayi, Taiwan; 5 Department of Nursing, Fooyin University, Kaohsiung, Taiwan; 6 Department of Information Management, National Central University, No. 300, Zhongda Rd, Zhongli District, Taoyuan City, 320317, Taiwan, 886 3 4227151 ext 66560; 7 Asian Institute for Impact Measurement and Management, National Central University, Taoyuan City, Taiwan

**Keywords:** atrial fibrillation, stroke, electronic medical records, text mining, clinical decision support

## Abstract

**Background:**

Stroke remains one of the leading causes of mortality and long-term disability worldwide. Atrial fibrillation (AF) is a major and often underdiagnosed risk factor for ischemic stroke as it is frequently asymptomatic and may remain undetected until a catastrophic cerebrovascular event occurs. The lack of timely identification and preventive treatment for AF substantially increases stroke risk. Although previous studies have proposed various predictive models for AF detection, many rely primarily on structured clinical variables and are developed using data from a single institution, which limits their generalizability and real-world applicability across different health care settings.

**Objective:**

The objective of this study was to develop a robust and generalizable AF risk prediction model for patients with stroke using electronic medical records. By integrating structured clinical variables with features derived from unstructured clinical text, this study aimed to construct a more comprehensive representation of patient health status. Furthermore, this study emphasized systematic internal and external validation, along with calibration assessment, to evaluate model stability and generalizability across multiple hospital datasets, thereby supporting its potential use in routine clinical practice.

**Methods:**

This study analyzed datasets from 2 hospitals in Taiwan: Landseed International Hospital (LIH), with 3988 patients, and Chia-Yi Christian Hospital (CYCH), with 5821 patients. We applied 5 feature engineering techniques to extract features from unstructured electronic medical record data, addressed data imbalance using 6 distinct resampling methods, and used 9 classification algorithms to compare model performance across both internal and external validation sets. This study identified the top 20 most important features from the best-performing models for both the LIH and CYCH datasets.

**Results:**

The optimal predictive model for LIH was based solely on structured variables, whereas the model for CYCH achieved superior results by integrating structured variables with text-derived variables obtained from unstructured clinical notes using term frequency–inverse document frequency. Notably, feature importance analysis consistently identified the ratio of E- to A-wave velocities, left atrial size, and age as the top 3 predictive factors across both datasets, underscoring their critical role in AF risk assessment among patients with stroke.

**Conclusions:**

This study demonstrated the development of predictive models for AF in patients with ischemic stroke. Notably, the integration of structured variables with variables derived from unstructured clinical text improved predictive performance in selected model configurations. Rigorous internal and external validation processes confirmed the superior performance of ensemble learning–based machine learning models compared with alternative algorithms, underscoring the potential of this approach for AF risk prediction.

## Introduction

Stroke ranks as the second leading cause of death and the third leading cause of disability worldwide, with both related mortality rates and the global disease burden continuing to rise [1,2]. The most common types of recurrent stroke are acute ischemic stroke (AIS) and transient ischemic attack (TIA) [3]. Within 1 year following an ischemic stroke, the risk of recurrence exceeds 7% [4-6]. Moreover, patients who experience a recurrent stroke within 10 years face more than twice the risk of death or disability compared with those who experience a first stroke [7].

Atrial fibrillation (AF) is a common sustained or paroxysmal cardiac arrhythmia [8,9]. Treatment may often be delayed as AF can be asymptomatic or episodic, making it challenging to detect [10]. AF is closely associated with cerebrovascular disease: irregular heartbeats over time can impair efficient blood contraction and clearance, potentially leading to blood clot formation. These clots may then travel to the brain and cause a stroke if AF remains untreated, increasing the risk of acute complications [11,12]. Patients with AF have twice the risk of dying from cardiovascular disease and up to a 5-fold increased risk of stroke [13]. Consequently, AF impacts patients with stroke significantly in terms of risk, treatment, and daily care requirements [14-17]. Regarding medication, for patients with stroke and AF, oral anticoagulants (eg, factor IIa and Xa inhibitors and warfarin) have been shown to reduce mortality by one-quarter and the risk of recurrent stroke by two-thirds [12]. Comparatively, antiplatelet drugs can reduce the risk of recurrent stroke by three-fifths [18]. Newer direct oral anticoagulants such as apixaban, rivaroxaban, edoxaban, and dabigatran are considered relatively safer and more effective, with a reduced risk of major bleeding compared with warfarin [19,20]. Physicians should find more underdiagnosed patients with AF, mostly paroxysmal AF, among those with AIS and TIA to administer appropriate safe and effective treatment for stroke prevention [21].

Risk assessment scales such as CHA2DS2-VASc [22] and its 2024 updated CHA2DS2-VA version [23] are widely used as clinical tools for evaluating AF risk based on variables collected from patient data. However, these assessments often require additional time or supplementary screening items to thoroughly identify associated risks. Long-term screening studies reveal that the diagnosis rate of AF, particularly paroxysmal AF, tends to increase with extended electrocardiographic (electrocardiogram; ECG) monitoring, although this also raises costs. Furthermore, collecting ECG data is time-intensive and can be cumbersome. Some researchers advocate for the use of continuous ECG monitoring (eg, Holter monitors) for AF detection, noting that recordings exceeding 24 hours may improve detection rates. However, given limited medical resources [7], the presence or absence of AF in patients with stroke significantly influences both treatment outcomes and subsequent risk levels. Thus, it is crucial to develop an efficient screening method that enables physicians to more accurately identify high-risk patients [10].

Electronic medical records (EMRs) contain rich structured and unstructured information that can be leveraged to support risk stratification and early detection of AF among patients with ischemic stroke [24]. While prior studies have demonstrated the potential of machine learning models for AF prediction, many have relied primarily on structured variables and data from a single institution, which may limit model generalizability and real-world applicability across different clinical settings [25-27]. In addition, the incremental value of incorporating unstructured clinical text into predictive models has not been systematically evaluated using rigorous external validation.

The aim of this study was to develop and validate a robust and generalizable machine learning model for predicting AF during hospitalization among patients with ischemic stroke using EMRs. Specifically, we sought to (1) integrate structured clinical variables with features derived from unstructured clinical text; (2) systematically evaluate multiple feature extraction methods, resampling strategies, and classification algorithms; and (3) assess model performance and calibration through rigorous internal and external validation across 2 independent hospital datasets. By emphasizing generalizability and real-world applicability, this study aimed to provide an effective decision support tool to facilitate early identification of high-risk patients and support clinical screening strategies for AF after stroke.

## Methods

### Study Population

This retrospective study was conducted using EMR datasets from 2 hospitals in Taiwan. The study population included all inpatients who were diagnosed with or suspected of having ischemic stroke by a physician during hospitalization, as well as those who presented with stroke-related symptoms. The dataset from Landseed International Hospital (LIH) covered the period from 2018 to 2022 and included a total of 3988 patients, while the dataset from Chia-Yi Christian Hospital (CYCH) spanned 2007 to 2020 and comprised 5821 patients. In total, 9809 patients were initially identified across the 2 hospitals.

Because the objective of this study was to develop predictive models for AF among patients with ischemic stroke without a prior history of AF, several exclusion criteria were applied. Consistent with prior studies, patients with documented AF prior to the index stroke were classified as having known AF before stroke and were excluded from model development. Known AF before stroke was defined as an AF diagnosis recorded in the medical history at the time of admission, indicating that AF had been identified through outpatient visits or cardiac monitoring before the current hospitalization. In addition, patients with AF detected on admission were excluded. AF detected on admission was identified based on the initial ECG, admission records, or documentation in the present illness or past medical history.

After applying the inclusion and exclusion criteria, the LIH dataset consisted of 1969 inpatients with unstructured clinical records, 2032 inpatients with structured clinical data, and 1226 inpatients with both structured and unstructured data. Similarly, the CYCH dataset included 3319 inpatients with unstructured data, 1441 inpatients with structured data, and 1072 inpatients with both data types.

Patients with both structured and unstructured data were defined as the intersecting cohort, representing overlapping patients across the 2 data sources. This intersecting cohort constituted the final analytic population used for model development and evaluation to ensure consistent feature availability across all included patients. Patients with data available in only 1 data source were not included in the final modeling analyses.

In the LIH intersecting cohort, 7% (86/1226) of the patients were identified as having AF during hospitalization following ischemic stroke. In contrast, in the CYCH intersecting cohort, 32.6% (350/1072) of the patients met the criteria for AF. Therefore, both cohorts exhibited class imbalance with notably different proportions of AF cases, which motivated the use of resampling strategies in subsequent model development.

### Ethical Considerations

The study protocol was approved by the institutional review boards of LIH (IRB-22-049) and Ditmanson Medical Foundation Chia-Yi Christian Hospital (IRB2022071). As this was a retrospective study using de-identified medical records, the requirement for informed consent was waived by both institutional review boards. To ensure patient confidentiality, all personal identifiers were removed and replaced with unique study identification numbers prior to analysis. No participants received any financial compensation, as no direct contact or intervention was involved.

### Outcome Variable

The primary outcome variable in this study was the occurrence of AF during hospitalization following ischemic stroke among patients in the intersecting cohort. AF status was determined using a predefined set of criteria that integrated multiple data sources from the EMRs, as summarized in Table 1. Specifically, AF was ascertained based on evidence from structured diagnostic information, unstructured clinical text, and medication records. Structured data included *International Classification of Diseases* diagnosis codes indicative of AF, whereas unstructured sources comprised textual mentions of AF in clinical narratives and examination reports, such as Holter monitoring reports; cardiac ultrasound reports; ECG reports, excluding the first ECG on admission; and general medical records, including discharge summaries. Keyword-based text searches were conducted using commonly used terms referring to AF. In addition, prescriptions of antiarrhythmic agents and oral anticoagulants were incorporated as supportive evidence for AF identification [28]. A patient was classified as having AF if any one of the criteria listed in Table 1 was satisfied.

**Table 1.** Criteria used to ascertain atrial fibrillation (AF) status in the intersecting cohort.

Criterion	Conditions
AF-1	AF documented in the stroke registry during hospitalization
AF-2	AF-related keywords identified in Holter monitoring report text
AF-3	AF-related keywords identified in cardiac ultrasound report text
AF-4	AF-related keywords identified in ECG^a^ report text, excluding the first ECG on admission
AF-5	AF-related keywords identified in clinical narratives, including hospital discharge summaries
AF-6	Presence of an AF-related *ICD*^b^ diagnosis code (*ICD-9-CM*^c^ 427.31 or *ICD-10-CM*^d^ I48.91)
AF-7	Prescription of antiarrhythmic medications, including amiodarone, propafenone, or dronedarone
AF-8	Prescription of oral anticoagulants, including warfarin, apixaban, edoxaban, rivaroxaban, or dabigatran

a ECG: electrocardiogram.

b *ICD*: *International Classification of Diseases*.

c *ICD-9-CM*: *ICD, Ninth Revision, Clinical Modification*.

d *ICD-10-CM*: *ICD, 10th Revision, Clinical Modification*.

### Predictor Variables and Text Preprocessing

Predictor variables comprised data obtained within 72 hours of admission. After excluding patients with a history of AF or diagnosed with AF upon admission, all variables were standardized across both hospitals.

The structured variables, detailed in Multimedia Appendix 1, comprised a comprehensive set of clinical predictors that are routinely available at hospital admission and during early hospitalization. These structured predictors included demographic characteristics (sex, age, height, and weight); vascular imaging findings derived from carotid duplex sonography; echocardiographic parameters reflecting cardiac structure and function (eg, aortic diameter, left atrial size, ventricular dimensions, ejection fraction, and diastolic function indexes); vital signs (body temperature, heart rate, respiratory rate, and blood pressure); and laboratory measurements covering hepatic function, renal function, coagulation profiles, inflammatory markers, lipid profiles, and hematological indexes. In addition, neurological severity was captured using individual National Institutes of Health Stroke Scale (NIHSS) subitems, as well as the overall NIHSS score, enabling fine-grained representation of stroke-related neurological deficits. Family medical history variables related to hypertension, diabetes mellitus, stroke, and ischemic heart disease were also included as nominal predictors. Together, these structured variables provided a multidimensional characterization of patients’ demographic profiles, cardiovascular status, metabolic conditions, neurological severity, and familial risk factors.

Unstructured data encompassed magnetic resonance imaging results, chief concerns, and present illness. Text preprocessing of variables derived from unstructured clinical text involved Chinese-English translation, spell-checking, abbreviation expansion, nonword symbol removal, AF-suggestive word deletion, lowercase conversion, word form reduction, negation marking as “_NEG” using additional negation words [29,30] and the Natural Language Toolkit’s (Team NLTK) *mark_negation* function, and stop word removal.

For text feature extraction, we used bidirectional encoder representations from transformers (BERT) [31,32], Doc2Vec [33,34], term frequency–inverse document frequency (TF-IDF) [35,36], and MetaMap [37,38]. These 4 methods were selected to represent complementary paradigms in clinical natural language processing, ranging from deep contextualized language models to traditional statistical and knowledge-based approaches. BERT has demonstrated strong performance in modeling contextual semantics and capturing complex linguistic patterns in clinical narratives and has been widely adopted in biomedical and clinical natural language processing tasks. Doc2Vec was included as a representative neural embedding method that captures document-level semantics with lower computational complexity, making it suitable for large-scale clinical text analysis. TF-IDF remains a widely used and robust baseline in clinical text mining due to its interpretability and effectiveness in high-dimensional sparse representations. MetaMap was incorporated to leverage domain-specific medical knowledge by mapping clinical text to standardized concepts in the Unified Medical Language System, which has been shown to enhance semantic normalization and downstream clinical prediction tasks.

Medical concepts (concept unique identifiers) were identified via MetaMap and subsequently transformed into feature vectors using TF-IDF applying a term frequency threshold with maximum and minimum values set to 0.9 and 0.1, respectively. In addition, Chinese text translation and spell-checking were conducted using *googletrans* (version 3.1.0a0) and *pyspellchecker* (version 0.8.1) to improve text consistency prior to feature extraction.

### Prediction Model Development and Evaluation

**Figure 1.**
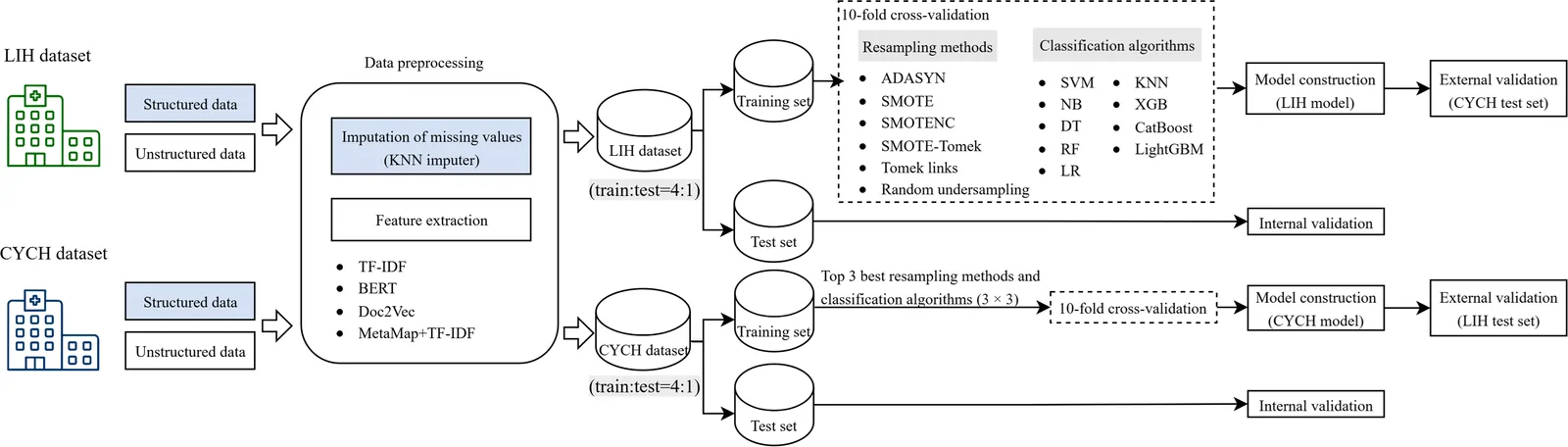
The process of machine learning model construction. ADASYN: adaptive synthetic sampling; BERT: bidirectional encoder representations from transformers; CatBoost: categorical boosting; CYCH: Chia-Yi Christian Hospital; DT: decision tree; KNN: k-nearest neighbor; LightGBM: light gradient-boosting machine; LIH: Landseed International Hospital; LR: logistic regression; NB: naive Bayes; RF: random forest; SMOTE: synthetic minority oversampling technique; SMOTENC: SMOTE for nominal and continuous features; SVM: support vector machine; TF-IDF: term frequency–inverse document frequency; XGB: extreme gradient boosting.

Figure 1 delineates a comprehensive machine learning model validation workflow encompassing both internal and external validation phases. The internal validation phase uses the LIH dataset and a 10-fold cross-validation strategy. During this process, various combinations of resampling methods and modeling techniques are evaluated, with the top 3 best-performing combinations (totaling 9 configurations) selected for further analysis. This step is crucial for identifying the most appropriate models and parameter settings for the given data.

The external validation phase leverages an independent dataset from CYCH. In this stage, the optimal model and resampling method combinations identified during internal validation are used to train models, which are subsequently evaluated on the external dataset. This phase is essential for assessing the models’ generalizability, ensuring their robustness and efficacy when applied to novel, unseen data. This rigorous 2-stage validation approach enhances the reliability and external validity of the developed predictive models, providing a robust framework for clinical application in AF risk assessment among patients with stroke. For both the internal and external validation phases, we used k-nearest neighbor imputation to address missing values within structured data. Additionally, we applied multiple feature extraction techniques to the unstructured text, including TF-IDF, BERT, Doc2Vec, and MetaMap combined with TF-IDF, followed by subsequent evaluations to compare model performance. To comprehensively evaluate the predictive power of different data types, we constructed and compared three distinct model configurations: (1) models using only structured variables, (2) models using only variables derived from unstructured clinical text (ie, text-derived variables), and (3) models combining both structured and text-derived variables.

To address the class imbalance present in the datasets from both hospitals, we implemented various resampling techniques during the model training phase, including adaptive synthetic sampling, synthetic minority oversampling technique (SMOTE), SMOTE for nominal and continuous features, SMOTE-Tomek, Tomek links, and random undersampling. We stratified and split the data into training and test sets with a 4:1 ratio using the parameter *stratify=df[[‘AF’]]* to maintain consistent class ratios across sets.

We evaluated multiple classifiers, including support vector machine, Gaussian naïve Bayes, k-nearest neighbor, decision tree, random forest, logistic regression, Extreme Gradient Boosting (XGBoost), categorical boosting (CatBoost), and light gradient-boosting machine (LightGBM). The training process incorporated 10-fold cross-validation with stratification (*stratify=df[[‘AF’]]*) to obtain robust performance estimates. We assessed model performance using multiple evaluation metrics, including the area under the receiver operating characteristic curve (AUC), sensitivity, specificity, and expected calibration error. Following the identification of optimal model combinations, we conducted both internal and external validation. The final results reflected the outcomes of the test set.

To enhance model interpretability, we used Shapley values (using *shap* version 0.45.0) to explain the best-performing models in both the LIH and CYCH datasets. This approach provides insights into the relative importance of different features in the prediction task.

### Baseline Models

The baseline model was compared with machine learning models using traditional risk-scoring models (AS5F [age, stroke severity NIHSS>5 to find atrial fibrillation] [39-41] and CHASE-LESS [coronary, heart failure, age, stroke severity–lipidemia, sugar, and prior stroke] [15,42,43]). CHASE-LESS was developed using data derived from a Taiwanese population, which is consistent with the population of this study, and its variables include age, NIHSS score, coronary heart disease, congestive heart failure, hyperlipidemia, diabetes mellitus, and prior stroke or TIA. The threshold value of CHASE-LESS was set to 6, and the scoring items were based on the variables in the EMR at the time of admission. The AS5F score is designed to determine the need for long-term ECG monitoring in patients with AIS or TIA.

## Results

We conducted modeling and prediction using both structured and text-derived variables from the two hospitals. In the internal validation for LIH (Table 2), the combination of structured variables with logistic regression yielded the best performance, achieving an AUC of 0.896. For text-derived variables, the model using Doc2Vec, random undersampling, and LightGBM achieved an AUC of 0.674. When integrating both structured and text-derived variables, the model incorporating Doc2Vec, SMOTE, and CatBoost performed well, with an AUC of 0.885. Some models showed enhanced performance when integrating both types of variables. For instance, in the LightGBM model implementation, the integration of structured and text-derived variables, coupled with the adaptive synthetic sampling technique, demonstrated superior performance, with an AUC of 0.876. This feature-integrated approach substantially outperformed both models using only structured variables (AUC=0.850) and those using only text-derived variables (AUC=0.468). However, not all combined approaches demonstrated such improvement.

**Table 2.** Comparative analysis of Landseed International Hospital (LIH) models for atrial fibrillation prediction for the area under the receiver operating characteristic curve (AUC) metric: internal validation using the LIH dataset.

Model	Structured variables, AUC	Text-derived variables, AUC				Structured+text-derived variables, AUC			
		BERT^a^	Doc2Vec	MetaMap	TF-IDF^b^	BERT	Doc2Vec	MetaMap	TF-IDF
AS5F^c^	0.597	—^d^	—	—	—	—	—	—	—
CHASE-LESS^e^	0.657	—	—	—	—	—	—	—	—
SVM^f^									
Imbalanced	0.835	0.403	0.496	0.499	0.64	0.654	0.837	0.809	0.862
Undersampling	0.778	0.579	0.61	0.48	0.595	0.482	0.76	0.779	0.841
ADASYN^g^	0.812	0.548	0.514	0.466	0.602	0.504	0.846	0.81	0.868
SMOTE^h^	0.803	0.54	0.511	0.51	0.672	0.504	0.824	0.783	0.796
Gaussian NB^i^									
Imbalanced	0.52	0.439	0.604	0.572	0.466	0.404	0.535	0.566	0.468
Undersampling	0.763	0.495	0.671	0.483	0.574	0.616	0.764	0.551	0.599
ADASYN	0.455	0.402	0.533	0.534	0.643	0.452	0.456	0.545	0.52
SMOTE	0.458	0.507	0.532	0.55	0.525	0.477	0.42	0.547	0.609
KNN^j^									
Imbalanced	0.618	0.463	0.47	0.519	0.666	0.486	0.642	0.58	0.567
Undersampling	0.646	0.398	0.451	0.491	0.527	0.526	0.576	0.628	0.648
ADASYN	0.627	0.529	0.45	0.483	0.506	0.421	0.603	0.691	0.625
SMOTE	0.594	0.446	0.427	0.511	0.548	0.405	0.577	0.582	0.608
DT^k^									
Imbalanced	0.542	0.529	0.512	0.535	0.503	0.613	0.576	0.531	0.553
Undersampling	0.621	0.433	0.509	0.49	0.539	0.673	0.634	0.501	0.531
ADASYN	0.666	0.439	0.584	0.521	0.448	0.723	0.631	0.567	0.675
SMOTE	0.545	0.45	0.599	0.574	0.439	0.713	0.673	0.604	0.548
RF^l^									
Imbalanced	0.815	0.548	0.535	0.533	0.586	0.627	0.758	0.757	0.756
Undersampling	0.846	0.376	0.658	0.553	0.523	0.609	0.838	0.738	0.829
ADASYN	0.82	0.523	0.532	0.487	0.476	0.6	0.832	0.79	0.821
SMOTE	0.86	0.502	0.539	0.522	0.565	0.621	0.845	0.815	0.851
LR^m^									
Imbalanced	0.896	0.523	0.659	0.504	0.641	0.798	0.862	0.819	0.805
Undersampling	0.84	0.462	0.664	0.492	0.558	0.769	0.783	0.805	0.827
ADASYN	0.865	0.463	0.635	0.537	0.621	0.752	0.847	0.789	0.804
SMOTE	0.866	0.471	0.624	0.543	0.644	0.757	0.833	0.803	0.787
XGB^n^									
Imbalanced	0.845	0.402	0.541	0.571	0.653	0.813	0.812	0.822	0.822
Undersampling	0.829	0.408	0.617	0.537	0.547	0.797	0.787	0.824	0.826
ADASYN	0.849	0.41	0.5	0.46	0.504	0.865	0.868	0.86	0.874
SMOTE	0.856	0.449	0.591	0.474	0.581	0.882	0.868	0.857	0.819
CatBoost^o^									
Imbalanced	0.869	0.41	0.609	0.517	0.652	0.774	0.85	0.778	0.849
Undersampling	0.868	0.407	0.657	0.536	0.573	0.772	0.854	0.806	0.835
ADASYN	0.864	0.524	0.522	0.481	0.558	0.809	0.875	0.857	0.864
SMOTE	0.855	0.46	0.567	0.529	0.564	0.833	0.885	0.84	0.866
LightGBM^p^									
Imbalanced	0.829	0.524	0.46	0.619	0.538	0.801	0.75	0.837	0.798
Undersampling	0.783	0.361	0.674	0.435	0.513	0.777	0.79	0.783	0.81
ADASYN	0.85	0.471	0.642	0.604	0.468	0.852	0.843	0.773	0.876
SMOTE	0.858	0.488	0.665	0.616	0.525	0.811	0.873	0.845	0.837

a BERT: bidirectional encoder representations from transformers.

b TF-IDF: term frequency–inverse document frequency.

c AS5F: age, stroke severity National Institutes of Health Stroke Scale >5 to find atrial fibrillation.

d Not applicable.

e CHASE-LESS: coronary, heart failure, age, stroke severity–lipidemia, sugar, and prior stroke.

f SVM: support vector machine.

g ADASYN: adaptive synthetic sampling.

h SMOTE: synthetic minority oversampling technique.

i NB: naïve Bayes.

j KNN: k-nearest neighbor.

k DT: decision tree.

l RF: random forest.

m LR: logistic regression.

n XGB: extreme gradient boosting.

o CatBoost: categorical boosting.

p LightGBM: light gradient-boosting machine.

In the external validation of the LIH model using the CYCH dataset (Table 3), the best performance was achieved using a combination of BERT and SMOTE, with an AUC of 0.795, which exceeded the AUC for models using only structured variables (AUC=0.781) and those using only text-derived variables. In the internal validation for CYCH (Table 4), the best-performing model was a combination of BERT with undersampling and XGBoost, achieving an AUC of 0.861. In the external validation, the optimal combination involved an imbalanced technique with CatBoost, resulting in an AUC of 0.832. These findings suggest that combining structured and text-derived variables can improve predictive performance in some models, highlighting the importance of selecting appropriate models and resampling techniques to enhance model outcomes.

**Figure 2.**
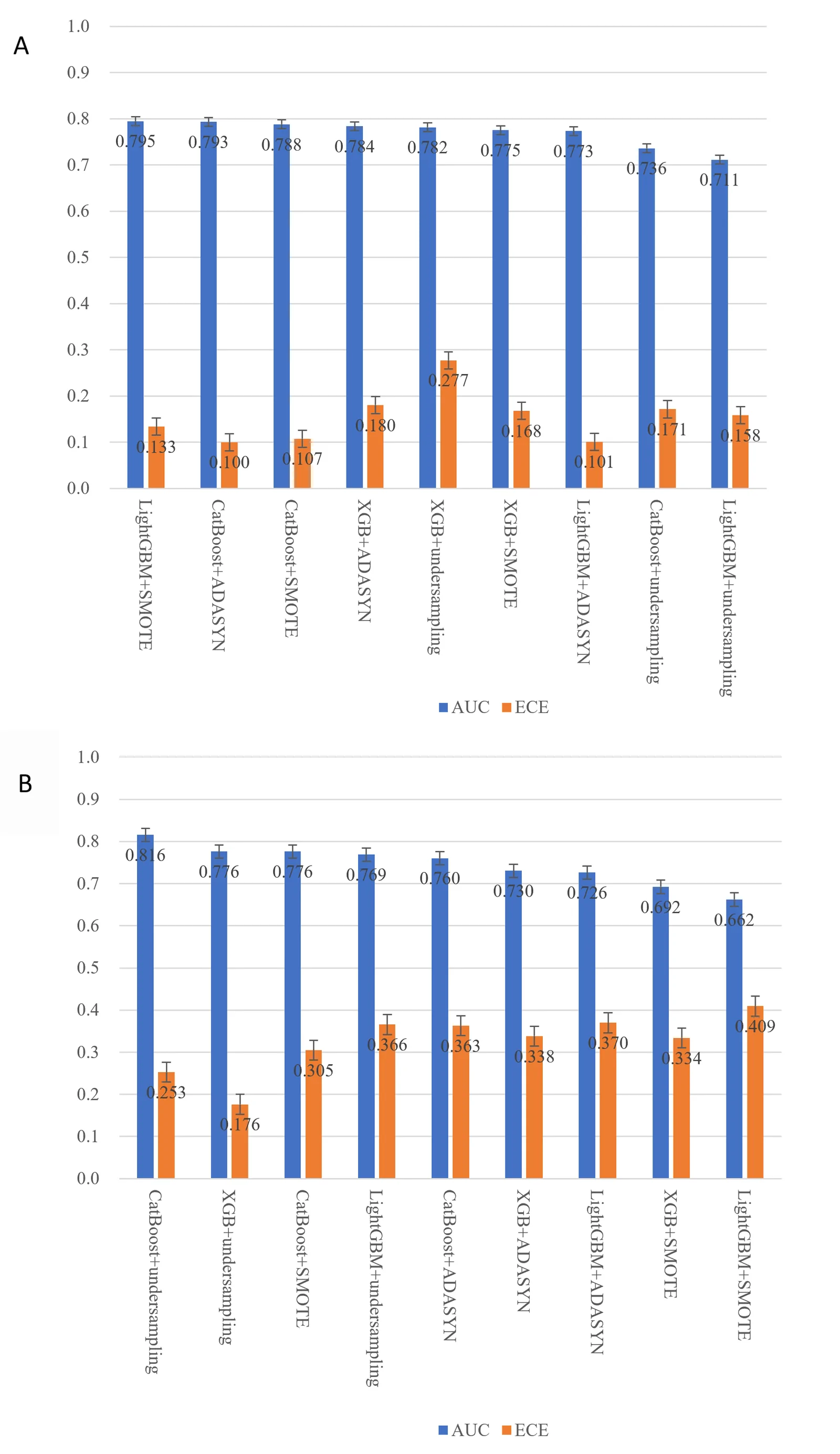
Ranking of the top 3 model and resampling method combinations for external validation: validation of the Landseed International Hospital (LIH) model using the Chia-Yi Christian Hospital (CYCH) dataset (A) and validation of the CYCH model using the LIH dataset (B). ADASYN: adaptive synthetic sampling; AUC: area under the receiver operating characteristic curve; CatBoost: categorical boosting; ECE: expected calibration error; LightGBM: light gradient-boosting machine; SMOTE: synthetic minority oversampling technique; XGB: extreme gradient boosting.

Figure 2 presents the external validation results from the 2 hospitals, indicating that the combination of CatBoost and SMOTE ranked third in both cases as determined by the AUC metric. Overall, ensemble learning demonstrated superior performance throughout the validation process. While the incorporation of text-derived variables did not consistently yield the highest AUC values in both internal and external validation, it is noteworthy that their inclusion generally resulted in improved outcomes based on the expected calibration error during external validation.

**Table 3.** Performance of the top 3 Landseed International Hospital models incorporating structured and unstructured feature combinations: external validation using the Chia-Yi Christian Hospital dataset.

Model	AUC^a^	Sensitivity	Specificity	ECE^b^
XGB^c^				
Structured	0.781	0.651	0.776	0.187
Structured+MRI^d^	0.771	0.549	0.843	0.174
Structured+CC^e^	0.776	0.623	0.796	0.171
Structured+PI^f^	0.773	0.597	0.798	0.178
Structured+MRI+CC+PI	0.775	0.571	0.821	0.168
CatBoost^g^				
Structured	0.789	0.463	0.892	0.09
Structured+MRI	0.781	0.311	0.942	0.147
Structured+CC	0.788	0.483	0.895	0.093
Structured+PI	0.79	0.46	0.91	0.104
Structured+MRI+CC+PI	0.788	0.443	0.911	0.107
LightGBM^h^				
Structured	0.781	0.611	0.81	0.161
Structured+MRI	0.794	0.509	0.889	0.111
Structured+CC	0.772	0.5	0.864	0.124
Structured+PI	0.784	0.509	0.873	0.124
Structured+MRI+CC+PI	0.795	0.574	0.855	0.133

a AUC: area under the receiver operating characteristic curve.

b ECE: expected calibration error.

c XGB: extreme gradient boosting.

d MRI: magnetic resonance imaging.

e CC: chief concern.

f PI: present illness.

g CatBoost: categorical boosting.

h LightGBM: light gradient-boosting machine.

**Table 4.** Performance of the top 3 Chia-Yi Christian Hospital (CYCH) models incorporating structured and unstructured (magnetic resonance imaging+chief concerns+present illness) feature combinations.

Model	Internal validation (CYCH dataset), AUC^a^	External validation (LIH^b^ dataset)	
		AUC	ECE^c^
XGB^d^			
Imbalanced	0.858	0.816	0.08
Undersampling	0.861	0.776	0.176
ADASYN^e^	0.852	0.73	0.338
SMOTE^f^	0.845	0.692	0.334
CatBoost^g^			
Imbalanced	0.851	0.832	0.129
Undersampling	0.859	0.816	0.253
ADASYN	0.843	0.76	0.363
SMOTE	0.843	0.776	0.305
LightGBM^h^			
Imbalanced	0.84	0.764	0.225
Undersampling	0.816	0.769	0.366
ADASYN	0.789	0.726	0.37
SMOTE	0.778	0.662	0.409

a AUC: area under the receiver operating characteristic curve.

b LIH: Landseed International Hospital.

c ECE: expected calibration error.

d XGB: extreme gradient boosting.

e ADASYN: adaptive synthetic sampling.

f SMOTE: synthetic minority oversampling technique.

g CatBoost: categorical boosting.

h LightGBM: light gradient-boosting machine.

## Discussion

### Summary of Main Findings

This study developed and validated machine learning–based prediction models for identifying AF during hospitalization among patients with ischemic stroke by leveraging EMRs from 2 independent hospitals. The main findings can be summarized in 3 points. First, models trained on structured clinical variables achieved strong predictive performance, and the integration of unstructured clinical text further improved discrimination and calibration in selected model configurations, particularly in the external validation setting. Second, ensemble learning methods, including CatBoost, XGBoost, and LightGBM, consistently outperformed traditional risk scores and conventional classifiers across internal and external validation. Third, feature importance analysis using SHAP revealed that echocardiographic parameters, including the ratio of E- to A-wave velocities, left atrial size, and age, were among the most influential predictors across both datasets. Together, these findings demonstrate the feasibility and generalizability of incorporating structured and unstructured EMR data to support early risk stratification for AF after ischemic stroke.

### Implications of Integrating Structured and Text-Derived Variables

Although the integration of structured variables with variables derived from unstructured clinical text improved predictive performance in several model configurations, the results in Table 2 indicate that such integration did not uniformly outperform models based solely on structured variables. This finding warrants careful interpretation rather than being viewed as a limitation of the proposed framework. In this study, structured clinical variables already encompassed well-established and highly discriminative predictors of AF, including echocardiographic parameters such as the ratio of E- to A-wave velocities, left atrial size, and age. When such strong predictors are present, models trained exclusively on structured variables may achieve near-optimal performance, leaving limited room for additional gains from text-derived variables.

Moreover, variables derived from unstructured clinical text are inherently high-dimensional and potentially noisy, particularly when generated using representation techniques such as TF-IDF or Doc2Vec. For certain classifiers that are sensitive to feature dimensionality or redundancy, the inclusion of text-derived variables may dilute the contribution of strong structured predictors, resulting in marginal or inconsistent performance gains. This phenomenon is consistent with prior observations in studies integrating clinical text with structured EMR data.

Importantly, our results suggest that the benefit of feature-level integration is highly model dependent. Ensemble learning–based algorithms, including LightGBM, XGBoost, and CatBoost, demonstrated a greater capacity to exploit complementary information from heterogeneous feature sources, particularly when combined with appropriate resampling strategies. In contrast, simpler or less flexible classifiers did not consistently benefit from the inclusion of text-derived variables. These findings indicate that integrating unstructured clinical text should be viewed as a complementary strategy whose effectiveness depends on the interaction among feature representation, classifier architecture, and imbalance-handling methods.

In addition, Table 2 indicates that resampling did not consistently improve performance for all classifiers as some models achieved comparable results when trained on the original imbalanced data. This finding suggests that the necessity of resampling is model dependent. Ensemble learning–based classifiers may inherently mitigate class imbalance effects, whereas other models benefit more substantially from resampling techniques. Therefore, resampling should be considered a robustness-oriented strategy rather than a mandatory preprocessing step.

### Significant Features

**Figure 3.**
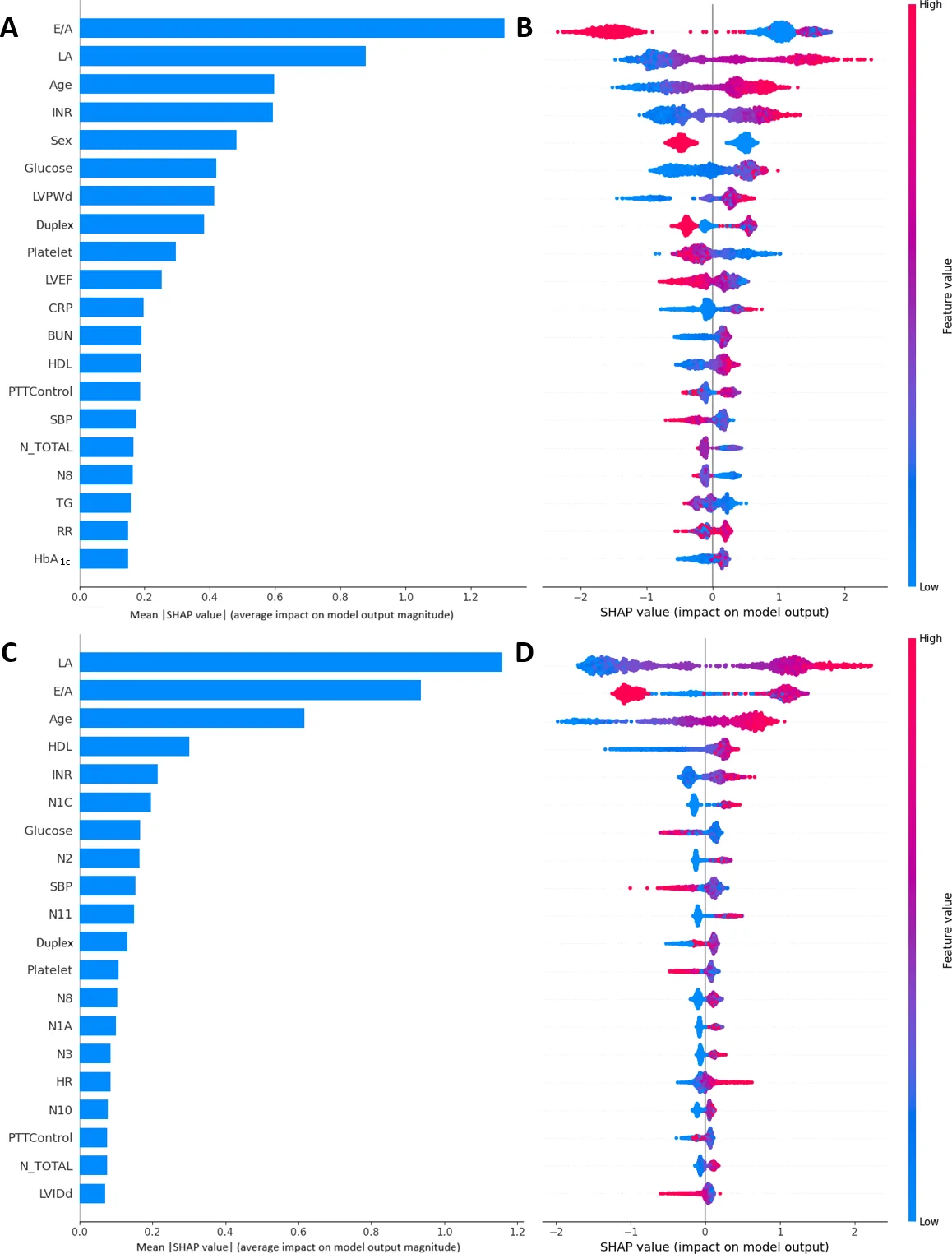
The top 20 most important features identified by the model based on structured variables. Panels A and C display bar charts representing the mean absolute Shapley values, which indicate the average contribution of each feature to the model’s output. Panels B and D present beeswarm plots of individual Shapley values for each feature across patients, where the position of each dot on the x-axis reflects that feature’s contribution to the prediction for a given patient. The color of each dot corresponds to the relative value of the associated feature. BUN: blood urea nitrogen; CRP: C-reactive protein; E/A: ratio of E- to A-wave velocities; HbA_1c_: hemoglobin A_1c_; HDL: high-density lipoprotein; HR: heart rate; INR: international normalized ratio; LA: left atrial size; LVEF: left ventricular ejection fraction; LVIDd: left ventricular internal dimension in diastole; LVPWd: left ventricular posterior wall in diastole; N1A: level of consciousness responsiveness; N1C: level of consciousness commands; N2: best gaze; N3: visual fields; N8: sensory; N10: dysarthria; N11: extinction and inattention; PTTControl: partial thromboplastin time control; RR: respiratory rate; SBP: systolic blood pressure; SHAP: Shapley additive explanations; TG: triglyceride.

Figure 3 illustrates the 20 most significant features identified for the LIH and CYCH datasets using shap (version 0.45.0). In Figure 3, features are ranked according to their mean absolute Shapley values, which quantify the global importance of each feature in relation to the model’s output. Figure 3A presents the feature ordering for LIH, whereas Figure 3C depicts the ordering for CYCH. Figure 3 shows beeswarm plots that visualize the Shapley values for each patient concerning these features. The beeswarm plots convey the contribution of each feature to the model’s output based on the magnitude and direction of the Shapley values. Figure 3B represents the contributions of LIH patients, whereas Figure 3D illustrates those of CYCH patients. Notably, the graphs reveal that the top 3 features consistently identified across both datasets were ratio of E- to A-wave velocities, left atrial size, and age.

### Clinical Applications and Significance

Predicting AF following a stroke is critical for effective secondary stroke prevention as it directly influences medical decision-making and patient outcomes. Given the constraints of health care resources, it is essential to optimize their use by incorporating efficient screening protocols. In this context, machine learning offers a highly effective approach. This study identified the optimal combination of variables derived from unstructured clinical text through rigorous external validation supplemented by internal validation using the LIH dataset and further validated using EMR data from CYCH. Although models built on internal data are often preferred for clinical applications, our experiments revealed that models developed using the CYCH data occasionally outperformed those based on LIH data. This discrepancy may be attributed to missing information and the relatively small sample size of the LIH dataset. The models developed in this study aim to support clinicians by identifying high-risk patients who would benefit from additional screening measures, such as ECG, ultimately reducing the risk of missed diagnoses and ensuring timely and appropriate treatment.

### Limitations

This study has several limitations that should be acknowledged. First, the data source poses a potential issue as the data were derived from EMRs within the hospitals. It is possible that patients were diagnosed with AF outside these institutions, introducing bias, particularly given the older age demographic of the hospital population. Second, regarding the data themselves, while generalizability was tested across 2 different hospitals, the variability in clerical styles between the hospitals may affect the consistency of the variables derived from unstructured clinical text. For structured variables, a high proportion of missing values in factors known to be associated with stroke or AF (eg, smoking history), uncertainties about the accuracy of the records, and the small and variable sample size could all impact the overall efficacy of the model. Finally, it should be noted that the predictors contributing to the model in this study do not imply causality.

### Conclusions

This study demonstrates the feasibility of developing machine learning–based predictive models to identify AF in patients with ischemic stroke using EMR data. The integration of structured variables with variables derived from unstructured clinical text, including medical history and magnetic resonance imaging findings extracted from free-text sources, showed potential to improve predictive performance in selected model configurations. Internal and external validation results indicate that ensemble learning–based models performed favorably compared with other algorithmic approaches, supporting their potential utility for AF risk prediction in clinical settings.

## Supplementary material

10.2196/78117 Multimedia Appendix 1 Detailed definitions and summary statistics of structured clinical variables extracted from electronic medical records at Landseed International Hospital and Chia-Yi Christian Hospital, including variable descriptions, data types, distributions, and proportions of missing values.

## Abbreviations

AF: atrial fibrillation
AIS: acute ischemic stroke
AS5F: age, stroke severity National Institutes of Health Stroke Scale>5 to find atrial fibrillation
AUC: area under the receiver operating characteristic curve
BERT: bidirectional encoder representations from transformers
CatBoost: categorical boosting
CHASE-LESS: coronary, heart failure, age, stroke severity–lipidemia, sugar, and prior stroke
CYCH: Chia-Yi Christian Hospital
ECG: electrocardiogram
EMR: electronic medical record
LightGBM: light gradient-boosting machine
LIH: Landseed International Hospital
NIHSS: National Institutes of Health Stroke Scale
SHAP: Shapley additive explanations
SMOTE: synthetic minority oversampling technique
TF-IDF: term frequency–inverse document frequency
TIA: transient ischemic attack
XGBoost: extreme gradient boosting
