# Correction: Enhanced Prediction of Atrial Fibrillation in Patients With Ischemic Stroke Through Electronic Medical Records and Text Mining: Algorithm Development and Validation

**DOI:** 10.2196/107705

**Published:** 2026-08-21

**Authors:** JMIR Publications Editorial Office

**Affiliations:** 1 JMIR Publications, 130 Queens Quay East, Suite 1100-1102Toronto, ON, Canada, 1 4165832040

Following the publication of “Enhanced Prediction of Atrial Fibrillation in Patients With Ischemic Stroke Through Electronic Medical Records and Text Mining: Algorithm Development and Validation” [1], concerns were raised regarding potential conflicts of interest between reviewers Szu-Yin Lin and Pei-Ju Lee and the authors. These concerns included prior co-publication with members of the author list. These conflicts were not disclosed to the JMIR Publications Editorial Office as per journal policy.

This article has been subsequently reassessed by the editor in chief, who determined that the content of these reviews did not unduly influence the decision to accept the article. The decision to accept the submission was based primarily on the editor in chief’s contemporary appraisal of the manuscript.

As such, the JMIR Publications Editorial Office issues this corrigendum to update the Conflicts of Interest statement from the following:

None declared.

The Conflicts of Interest statement now reads:

Reviewers Szu-Yin Lin and Pei-Ju Lee were found to have undisclosed conflicts of interest with members of the author list under the terms of JMIR Publications policy. The editor in chief determined that the content of the reviews did not unduly influence the decision to accept the article. The decision to accept the submission was based primarily on the editor in chief’s contemporary appraisal of the manuscript.

We regret that these issues were not identified before publication.

The correction will appear in the online version of the paper on the JMIR Publications website together with the publication of this correction notice. Because this was made after submission to PubMed, PubMed Central, and other full-text repositories, the corrected article has also been resubmitted to those repositories.
