# Supplementary material for: Enhanced Prediction of Atrial Fibrillation in Patients With Ischemic Stroke Through Electronic Medical Records and Text Mining: Algorithm Development and Validation
Source: JMIR Med Inform. 2026 Mar 10;14:e78117. doi: 10.2196/78117 (PMC12975001; doi:10.2196/78117)
Supplement: Multimedia Appendix 1 [file medinform-v14-e78117-s001.docx]

***Multimedia Appendix 1***

Table S1 outlines the common structured variables of LIH and CYCH, including data types and proportions of missing values.

**Table S1.** Clinical features of the study population.

| Variable Name | Description | Data type | LIH | CYCH | LIH  missing | CYCH  missing |
| --- | --- | --- | --- | --- | --- | --- |
| **Demographic characteristics** | | | | | | |
| Sex | Male, n (%) | Nominal | 809 (66.0) | 604 (56.3) | 0.0% | 0.0% |
| Age | Age, years, mean (SD^a^) | Numeric | 66.1 (14.0) | 67.9 (14.9) | 0.0% | 0.0% |
| Height | Height, mean (SD) | Numeric | 161.2 (8.8) | 160.0 (9.1) | 0.0% | 0.0% |
| Weight | Weight, mean (SD) | Numeric | 65.7 (13.5) | 63.1 (13.9) | 0.0% | 0.0% |
| **Vascular imaging findings** | | | | |  |  |
| Duplex | Detection of carotid atherosclerotic disease by carotid duplex sonography, True | Nominal | 673 (54.9) | 300 (28.0) | 13.1% | 50.2% |
| **Echocardiographic parameters, mean (SD)** | | | | | | |
| AO | Aorta | Numeric | 31.6 (4.1) | 32.2 (4.6) | 0.0% | 3.3% |
| IVS | Interventricular septum | Numeric | 10.6 (2.7) | 11.9 (2.6) | 0.1% | 0.0% |
| LA | Left atrium | Numeric | 40.1 (7.0) | 39.3 (7.7) | 0.0% | 0.5% |
| LVIDd | Left ventricular internal dimension in diastole | Numeric | 49.6 (7.1) | 46.3 (7.5) | 0.1% | 0.2% |
| LVEF | Left ventricular ejection fraction | Numeric | 69.3 (9.7) | 65.9 (10.8) | 0.1% | 1.3% |
| LVIDs | Left ventricular internal dimension in systole | Numeric | 30.6 (6.4) | 29.3 (6.8) | 0.1% | 5.4% |
| LVPWd | Left ventricular posterior wall in diastole | Numeric | 9.7 (4.3) | 11.3 (2.2) | 0.1% | 0.0% |
| E/A | Ratio of E- to A-wave velocities, n (%) | Nominal | 836 (68.2) | 542 (50.6) | 5.6% | 33.3% |
| **Vital signs (SD)** | | | | | | |
| BT | Body temperature | Numeric | 36.3 (0.6) | 36.4 (0.7) | 54.7% | 0.0% |
| HR | Heart rate | Numeric | 77.6 (15.4) | 84.6 (20.3) | 7.6% | 0.0% |
| RR | Respiratory rate | Numeric | 18.8 (3.5) | 19.7 (2.0) | 14.9% | 0.0% |
| SBP | Systolic blood pressure | Numeric | 149.6 (25.8) | 161.4 (31.5) | 2.9% | 0.0% |
| DBP | Diastolic blood pressure | Numeric | 87.5 (17.4) | 92.2 (18.9) | 2.9% | 0.0% |
| **Laboratory measurements (SD)** | | | | | | |
| GPT | Glutamic pyruvic transaminase | Numeric | 25.0 (31.7) | 28.7 (38.2) | 0.1% | 7.3% |
| PTT | Partial thromboplastin time | Numeric | 27.6 (5.2) | 28.0 (4.7) | 0.1% | 1.0% |
| PTTControl | Partial thromboplastin time control | Numeric | 28.8 (0.9) | 29.4 (2.5) | 0.1% | 10.5% |
| GOT | Glutamic oxaloacetic transaminase | Numeric | 26.1 (44.3) | 31.2 (41.6) | 0.1% | 26.4% |
| BUN | Blood urea nitrogen | Numeric | 20.4 (12.4) | 19.9 (10.4) | 0.1% | 2.4% |
| Creatinine | Creatinine | Numeric | 1.2 (1.1) | 1.2 (0.9) | 0.1% | 0.56% |
| CRP | C-reactive protein | Numeric | 1.1 (2.8) | 4.7 (6.6) | 0.1% | 71.55% |
| Glucose | Glucose | Numeric | 153.5 (87.7) | 156.8 (77.5) | 0.1% | 0.0% |
| HbA1c | Glycated hemoglobin | Numeric | 6.9 (2.0) | 7.1 (1.9) | 0.1% | 37.97% |
| HDL | High-density lipoprotein | Numeric | 45.8 (12.5) | 47.0 (13.6) | 0.2% | 8.2% |
| Hct | Hematocrit | Numeric | 41.8 (5.8) | 40.6 (5.8) | 0.1% | 0.0% |
| Hb | Hemoglobin | Numeric | 14.0 (2.1) | 13.7 (2.2) | 0.1% | 0.0% |
| LDL | Low-density lipoprotein | Numeric | 117.9 (38.3) | 114.0 (39.3) | 0.2% | 8.30% |
| INR | International normalized ratio | Numeric | 1.0 (0.1) | 1.1 (0.3) | 0.1% | 9.1% |
| Platelet | Platelet | Numeric | 238.1 (81.8) | 209.4 (79.2) | 0.1% | 0.0% |
| TG | Triglyceride | Numeric | 146.2 (103.3) | 124.3 (101.2) | 10.2% | 2.8% |
| WBC | White blood cells | Numeric | 8.7 (3.1) | 8.6 (3.6) | 0.1% | 0.0% |
| **Neurological severity (NIHSS), mean (SD)** | | | | | | |
| N1A | Level of consciousness responsiveness | Numeric | 0.2 (0.5) | 0.4 (0.8) | 64.3% | 0.1% |
| N1B | Level of consciousness questions | Numeric | 0.2 (0.5) | 0.6 (0.9) | 64.3% | 0.1% |
| N1C | Level of consciousness commands | Numeric | 0.1 (0.5) | 0.4 (0.8) | 64.3% | 0.1% |
| N2 | Best gaze | Numeric | 0.1 (0.4) | 0.4 (0.7) | 64.3% | 0.1% |
| N3 | Visual fields | Numeric | 0.2 (0.5) | 0.5 (0.8) | 64.3% | 0.1% |
| N4 | Facial palsy | Numeric | 0.6 (0.7) | 0.8 (0.8) | 64.3% | 0.0% |
| N5AL | Motor arm (left) | Numeric | 0.7 (1.1) | 1.1 (1.4) | 64.3% | 0.1% |
| N5BR | Motor arm (right) | Numeric | 0.7 (1.1) | 1.0 (1.4) | 64.3% | 0.1% |
| N6AL | Motor leg (left) | Numeric | 0.6 (1.0) | 1.1 (1.4) | 64.3% | 0.1% |
| N6BR | Motor leg (right) | Numeric | 0.6 (1.0) | 1.0 (1.4) | 64.3% | 0.1% |
| N7 | Limb ataxia | Numeric | 0.6 (0.8) | 0.5 (0.7) | 64.3% | 0.1% |
| N8 | Sensory | Numeric | 0.3 (0.5) | 0.5 (0.6) | 64.3% | 0.1% |
| N9 | Best language | Numeric | 0.3 (0.7) | 0.7 (1.1) | 64.3% | 0.1% |
| N10 | Dysarthria | Numeric | 0.5 (0.6) | 0.8 (0.8) | 64.3% | 0.1% |
| N11 | Extinction and inattention | Numeric | 0.1 (0.4) | 0.2 (0.6) | 64.3% | 0.1% |
| N_TOTAL | NIHSS overall score | Numeric | 5.9 (6.6) | 10.0 (9.4) | 64.3% | 0.0% |
| **Family medical history, n (%)** | | | | | | |
| FatherMotherHT | Parents’ history of hypertension, true | Nominal | 300 (24.5) | 694 (64.7) | 8.7% | 0.0% |
| BrotherSisterHT | Siblings’ history of hypertension, true | Nominal | 71 (5.8) | 617 (57.6) | 8.9% | 0.0% |
| FatherMotherDM | Parents’ history of diabetes mellitus, true | Nominal | 198 (16.2) | 567 (52.9) | 8.7% | 0.0% |
| BrotherSisterDM | Siblings’ history of diabetes mellitus, true | Nominal | 49 (4.0) | 576 (53.7) | 8.9% | 0.0% |
| FatherMotherStroke | Parents’ history of stroke, true | Nominal | 92 (7.5) | 473 (44.1) | 8.7% | 0.0% |
| BrotherSisterStroke | Siblings’ parents’ history of stroke, true | Nominal | 34 (2.8) | 471 (43.9) | 9.0% | 0.0% |
| FatherMotherIHD | Parents’ history of ischemic heart disease, true | Nominal | 49 (4.0) | 383 (35.7) | 8.7% | 0.0% |
| BrotherSisterIHD | Siblings’ history of ischemic heart disease, true | Nominal | 12 (1.0) | 425 (39.7) | 8.9% | 0.0% |

^a^SD = standard deviation.
